# Association Between Phase Angle, Muscle Mass Distribution, and Quality of Life in Patients with Chronic Obstructive Pulmonary Disease

**DOI:** 10.3390/jcm15103839

**Published:** 2026-05-16

**Authors:** Lyazat Ibrayeva, Irina Bacheva, Malika Sadibekova

**Affiliations:** The Department of Internal Medicine, Karaganda Medical University, Karaganda 100017, Kazakhstan; ibraeva@qmu.kz (L.I.); bacheva@qmu.kz (I.B.)

**Keywords:** COPD, phase angle, bioelectrical impedance analysis, quality of life, body composition, sarcopenia, visceral adiposity, ECW/TBW ratio

## Abstract

**Background**: Chronic obstructive pulmonary disease (COPD) is associated with systemic alterations in body composition, including muscle mass loss and fat redistribution, which may influence patient-reported outcomes. However, the independent contribution of bioimpedance-derived parameters, particularly phase angle, to quality of life (QoL) remains unclear. **Methods**: This exploratory pilot study included 75 clinically stable patients with moderate-to-severe COPD (GOLD stages II–III). Body composition was assessed using segmental multi-frequency bioelectrical impedance analysis with the InBody 770 system. Evaluated parameters included fat-free mass (FFM), skeletal muscle mass (SMM), percent body fat (PBF), visceral fat area (VFA), extracellular water-to-total body water ratio (ECW/TBW), bone mineral content (BMC), and phase angle (PhA). Quality of life was assessed using the WHOQOL-BREF questionnaire. Associations between body composition parameters and QoL domains were analyzed using Spearman correlation analysis and multivariable linear regression models. **Results**: Despite a median body mass index (BMI) within the normal range (23.4 kg/m^2^), body fat mass exceeded reference values in both men and women. Fat-free mass and skeletal muscle mass were located near the lower range of expected values. Correlation analysis demonstrated predominantly weak associations between body composition parameters and QoL domains. Significant positive correlations were identified between the psychological QoL domain and fat-free mass (ρ = 0.238, *p* = 0.041), skeletal muscle mass (ρ = 0.240, *p* = 0.040), basal metabolic rate (ρ = 0.236, *p* = 0.043), and bone mineral content (ρ = 0.249, *p* = 0.033). In multivariable regression models, fat-free mass and skeletal muscle mass demonstrated consistent positive associations with both physical and psychological QoL domains. Whole-body and segmental phase angle parameters did not demonstrate significant associations with QoL outcomes. **Conclusions**: In patients with COPD, BMI alone may inadequately reflect underlying alterations in body composition. Muscle-related parameters, particularly fat-free mass and skeletal muscle mass, demonstrated more consistent associations with physical and psychological aspects of quality of life than obesity-related indicators. These findings suggest that bioelectrical impedance analysis may provide additional clinically relevant information beyond BMI when assessing body composition and quality of life in patients with COPD.

## 1. Introduction

Chronic obstructive pulmonary disease (COPD), as a multifactorial and heterogeneous disorder characterized by both pulmonary and extrapulmonary manifestations, remains a major global public health problem because of its high prevalence (approximately 10% of the adult population worldwide), increasing incidence associated with population aging, and substantial mortality burden, ranking as the third leading cause of death globally [1,2,3].

The systemic consequences of COPD contribute to the development of cardiovascular comorbidities, muscle atrophy, and osteoporosis [4], as well as impaired nutritional status and alterations in body composition [5,6,7,8]. These changes lead to physical inactivity and deconditioning and exert a significant negative impact on prognosis, including an increased risk of COPD exacerbations, depression, and mortality. According to previous studies, 30–60% of patients with COPD suffer from malnutrition [9,10], 20–40% demonstrate reduced muscle mass [5,6], and 15–21.6% present with sarcopenia [11,12]. As physical activity declines, muscle mass progressively decreases, and oxygen utilization becomes less efficient, resulting in a vicious cycle of deteriorating physical performance. Factors contributing to reduced muscle strength and endurance in these patients include chronic systemic inflammation, oxidative stress, physical inactivity, hypoxia, hormonal disturbances, nutritional deficiencies such as inadequate protein and vitamin D intake, and the use of systemic corticosteroids [13]. These processes directly affect health-related quality of life in patients with COPD [4]. Furthermore, sarcopenia has been associated with an increased frequency of falls, hospitalizations, and mortality and negatively influences clinical outcomes [9]. Both malnutrition and sarcopenia reduce exercise tolerance, increase the severity of pulmonary dysfunction, raise the likelihood of hospitalization, and worsen quality of life [6,11,14,15].

As COPD progresses, patients frequently experience reductions in fat-free mass, particularly skeletal muscle mass, contributing to respiratory and peripheral muscle dysfunction, decreased endurance, and more severe clinical manifestations [16]. At the same time, a subset of patients develops increased visceral adiposity, which is associated with metabolic dysregulation, insulin resistance, and systemic inflammation [17], thereby contributing to an elevated risk of cardiovascular and metabolic complications [18]. Importantly, disturbances in body composition in COPD are driven by underlying pathophysiological mechanisms that directly affect functional capacity and overall well-being, ultimately impairing quality of life.

Impaired quality of life is observed in virtually all chronic diseases [19]; however, patients with COPD frequently report poorer outcomes compared with individuals affected by other chronic conditions such as diabetes mellitus, hypertension, and chronic kidney disease [20].

Thus, despite growing interest in bioimpedance-derived parameters, their independent clinical significance in COPD remains uncertain, particularly after adjustment for key demographic and clinical factors. Specifically, it remains unclear whether markers reflecting cellular integrity, such as phase angle, or indicators reflecting body composition, such as fat-free mass and visceral adiposity, are more closely associated with patient-reported outcomes. Therefore, a comprehensive evaluation of patients with COPD requires assessment of the complex relationships among body composition, fluid balance, metabolic status, and quality of life.

The aim of the present study was to investigate the association between bioelectrical impedance analysis-derived body composition parameters, including phase angle, fat and muscle distribution, and bone mineral content, and quality of life in patients with moderate-to-severe COPD. Particular attention was given to the physical domain of quality of life, as well as the potential influence of phenotype-specific body composition characteristics. Given the cross-sectional design of the study, these analyses were considered exploratory and hypothesis-generating rather than intended to establish causal relationships or definitive prognostic models.

## 2. Materials and Methods

### 2.1. Study Design and Participants

An exploratory cross-sectional pilot study was conducted from 1 January 2024 to 31 December 2024. A total of 75 patients aged 40–75 years with confirmed chronic obstructive pulmonary disease (COPD), GOLD stages II–III, who were in a clinically stable condition and had not previously participated in pulmonary rehabilitation programs, were included in the study. Participants were selected using a random sampling approach from an outpatient COPD registry. The study population consisted of 63 men and 12 women. All participants received standard pharmacological therapy in accordance with current clinical guidelines. Spirometric parameters, including forced expiratory volume in one second (FEV_1_), forced vital capacity (FVC), and the FEV_1_/FVC ratio, were obtained from medical records and are presented in Table 1. All participants provided written informed consent prior to study enrollment.

### 2.2. Body Composition Assessment

Body composition was assessed using the InBody 770 (InBody Co., Seoul, Republic of Korea), a multi-frequency segmental bioelectrical impedance analyzer based on the direct segmental multi-frequency bioelectrical impedance analysis (DSM-BIA) method. The device applies electrical currents at multiple frequencies (5, 50, 250, and 500 kHz) across five body segments (right arm, left arm, right leg, left leg, and trunk), allowing direct segmental measurements without the use of population-specific correction equations.

Measurements were performed under standardized conditions. Participants were examined in a fasting state and were instructed to avoid vigorous physical activity and excessive fluid intake for at least 2 h prior to testing.

The following parameters were recorded: phase angle (PhA, 50 kHz, whole body), extracellular water-to-total body water ratio (ECW/TBW), fat-free mass (FFM, kg), skeletal muscle mass (SMM, kg), visceral fat area (VFA, cm^2^), bone mineral content (BMC, kg), and percent body fat (PBF, %). All measurements were performed by trained personnel according to the manufacturer’s protocol.

### 2.3. Quality of Life Assessment

Quality of life was assessed using the WHOQOL-BREF questionnaire [21], a validated 26-item instrument widely used in clinical and epidemiological studies. Four domains were analyzed: physical health (QoL1), psychological health (QoL2), social relationships (QoL3), and environmental conditions (QoL4). For clarity and consistency throughout the manuscript, these WHOQOL-BREF quality-of-life profiles are hereafter referred to as “domains”. Higher scores indicated better perceived quality of life. Questionnaires were self-administered under researcher supervision to minimize missing data and response errors.

### 2.4. Statistical Analysis

Statistical analysis was performed using IBM SPSS Statistics Version 26. During the study planning stage, the required sample size was estimated using statistical power analysis for multiple linear regression. Assuming a medium effect size (f^2^ = 0.15), a significance level of α = 0.05, and statistical power of 80%, the minimum required sample size was calculated as 68 participants. A conservative increase in sample size was applied to account for potential exclusions. Considering the cross-sectional design and the minimal amount of missing data, the final sample of 75 participants was considered sufficient to maintain the target statistical power.

The distribution of continuous variables was assessed using the Shapiro–Wilk test. Since most variables demonstrated a non-normal distribution, data are presented as median and interquartile range [Me (Q1; Q3)]. Variables with normal distribution are presented as mean ± standard deviation (SD).

Associations between bioimpedance parameters and quality-of-life domains assessed using the WHOQOL-BREF scale were evaluated using Spearman’s rank correlation coefficient.

To identify independent associations between body composition parameters and quality-of-life outcomes, multivariable linear regression analysis was performed.

Considering the high conceptual and statistical overlap between fat-free mass (FFM) and skeletal muscle mass (SMM), with FFM including skeletal muscle as a major component and potentially dominating model effects, two separate modeling strategies were applied to improve the robustness and interpretability of the results:

- Model 1 included fat-free mass (FFM), basal metabolic rate (BMR), visceral fat area (VFA), and bone mineral content (BMC);
- Model 2 included skeletal muscle mass (SMM), basal metabolic rate (BMR), visceral fat area (VFA), and bone mineral content (BMC).

This approach allowed evaluation of the independent contribution of muscle-related parameters while minimizing the influence of multicollinearity.

Multicollinearity was assessed using variance inflation factors (VIFs), with values > 5 considered indicative of potential collinearity. Given the relatively small sample size and number of predictors, the multivariable models were considered exploratory. A two-sided *p*-value < 0.05 was considered statistically significant.

## 3. Results

The demographic, clinical, and body composition characteristics of the study population are presented in Table 1.

In the study population, the median BMI was within the normal reference range (23.4 kg/m^2^). However, body fat percentage exceeded sex-specific reference ranges in both men (median 24.7%) and women (median 38.7%), with a more pronounced elevation observed among women. Fat-free mass (median 51.4 kg) and skeletal muscle mass (median 28.3 kg) were located near the lower range of expected values, whereas overall body fat percentage (median 26.6%) approached the upper limits of normal.

The ECW/TBW ratio was located near the upper boundary of the reference interval (median 0.386). Whole-body phase angle (PhA) in men was 5.2° (4.7–5.8), which falls within the reference range (~5.0–7.0) but is positioned closer to its lower boundary. In women, whole-body PhA was 4.6° (4.2–5.1), also within the reference range (~4.5–6.5) but similarly located near the lower limit.

Segmental PhA values varied across body regions, with the highest values observed in the trunk (median 7.3°), whereas the lowest values were detected in the lower extremities (median 4.8°).

### 3.1. Correlation Analysis

**Figure 1:**
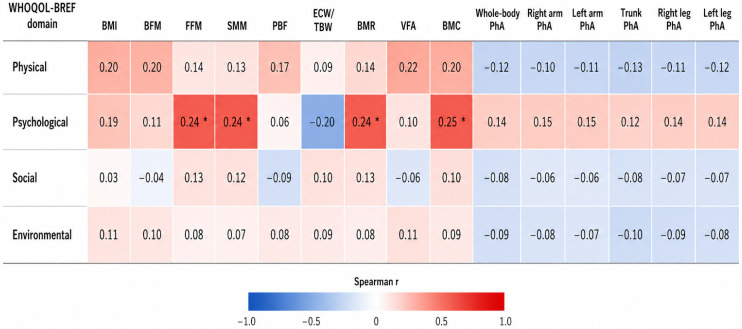
Spearman correlation analysis between the four quality-of-life domains assessed by the WHOQOL-BREF and bioimpedance-derived body composition parameters in patients with COPD. *p* < 0.05 (two-tailed), *—indicates statistically significant results. WHOQOL-BREF—World Health Organization Quality of Life Assessment–Brief Version; BMI—body mass index; BFM—body fat mass; FFM—fat-free mass; SMM—skeletal muscle mass; PBF—percent body fat; ECW/TBW—extracellular water to total body water ratio; BMR—basal metabolic rate; VFA—visceral fat area; BMC—bone mineral content; PhA—phase angle.

The results of the Spearman correlation analysis between the four quality-of-life domains assessed by the WHOQOL-BREF and bioimpedance-derived body composition parameters are presented in Figure 1. Detailed correlation results are presented in Supplementary Table S1.

Statistically significant positive correlations were observed only within the psychological domain, including fat-free mass (FFM) (ρ = 0.238, *p* = 0.041), skeletal muscle mass (SMM) (ρ = 0.240, *p* = 0.040), basal metabolic rate (BMR) (ρ = 0.236, *p* = 0.043), and bone mineral content (BMC) (ρ = 0.249, *p* = 0.033).

A trend toward positive correlations was identified between the physical health domain and BMI (ρ = 0.199, *p* = 0.089), body fat mass (ρ = 0.201, *p* = 0.085), visceral fat area (VFA) (ρ = 0.223, *p* = 0.056), and bone mineral content (ρ = 0.202, *p* = 0.085), although these associations did not reach statistical significance. In addition, the psychological domain demonstrated a tendency toward a negative correlation with the extracellular water-to-total body water ratio (ECW/TBW) (ρ = −0.196, *p* = 0.094).

No statistically significant correlations were identified in the social relationships or environmental domains. Whole-body and segmental phase angle (PhA) parameters also did not demonstrate significant associations with any quality-of-life domain.

### 3.2. Multivariable Linear Regression Analysis

Multivariable linear regression models were constructed for each WHOQOL-BREF domain to evaluate associations with body composition parameters. Fat-free mass, skeletal muscle mass, basal metabolic rate, visceral fat area, and bone mineral content were included as independent predictors. Considering the conceptual overlap between fat-free mass and skeletal muscle mass, separate models were developed to minimize multicollinearity (Table 2).

Among the investigated body composition parameters, statistically significant associations in the multivariable linear regression models were identified only for the physical and psychological domains of the WHOQOL-BREF. In both domains, fat-free mass (FFM) and skeletal muscle mass (SMM) emerged as significant positive predictors.

Within the physical health domain, significant effects were observed for FFM (B = 0.10, β = 0.35, *p* = 0.031) and SMM (B = 0.12, β = 0.33, *p* = 0.036). Similar associations were identified in the psychological domain, where both FFM (B = 0.11, β = 0.34, *p* = 0.029) and SMM (B = 0.14, β = 0.36, *p* = 0.026) demonstrated statistically significant positive relationships.

Bone mineral content (BMC) demonstrated a statistically significant positive association only with the psychological domain of the WHOQOL-BREF, both in the model including FFM (B = 1.02, β = 0.29, *p* = 0.041) and in the model including SMM (B = 1.05, β = 0.30, *p* = 0.038).

No statistically significant associations were identified in the remaining two domains (Social Relationships and Environment). Visceral fat area (VFA) and basal metabolic rate (BMR) did not reach statistical significance in the regression models, although BMR demonstrated a tendency toward positive associations in both the physical and psychological domains.

## 4. Discussion

In the present study, the median BMI of patients with COPD was within the normal body weight range, whereas percent body fat (PBF) was elevated in both men and women. This finding is of considerable clinical interest. On the one hand, numerous studies have demonstrated the adverse prognostic impact of low BMI on survival in patients with COPD [22,23]. On the other hand, several reports have described the so-called “obesity paradox”, whereby overweight and obesity are associated with better outcomes compared with normal or reduced BMI [23,24]. However, this concept should be interpreted with caution, as nutritional assessment based solely on BMI may mask the presence of sarcopenic obesity—a condition characterized by reduced muscle mass combined with excess adipose tissue accumulation, particularly visceral fat, despite normal or elevated BMI values [7,11,25,26]. Such body composition abnormalities are associated with more pronounced systemic inflammation, reduced physical tolerance, impaired quality of life, and unfavorable clinical outcomes, even in the presence of apparently normal BMI values.

Fat-free mass (FFM) and skeletal muscle mass (SMM) demonstrated statistically significant associations with both the physical and psychological domains of the WHOQOL-BREF in both correlation and regression analyses. These findings suggest that reductions in fat-free and/or muscle mass may contribute to deterioration in both physical and psychological aspects of quality of life [27]. This may be explained by the fact that muscle depletion is frequently accompanied by reduced functional capacity, lower exercise tolerance, impaired endurance, and limitations in daily activities [28]. Such changes directly affect the physical domain of quality of life, which includes perceptions of energy, mobility, work capacity, pain or discomfort, and dependence on external assistance.

At the same time, the observed association with the psychological domain suggests that alterations in body composition may have a broader clinical context. Reduced muscle and fat-free mass may be associated with perceived physical weakness [29], loss of independence [30], decreased confidence in one’s physical abilities, and poorer subjective health perception [31]. In turn, these factors may negatively influence psychological well-being, emotional status, and overall satisfaction with quality of life [32].

Interestingly, parameters reflecting fat mass volume and adiposity percentages did not demonstrate significant associations with quality of life, whereas associations were identified specifically for fat-free and muscle-related parameters. A similar inconsistency was observed for phase angle (PhA). Although PhA is frequently described in the literature as an integrative marker of cellular mass, nutritional status, functional reserve, and overall prognosis [33], no such association was identified in the present study. It is possible that, in patients with COPD, subjective quality of life is more strongly determined by preservation of muscle mass, which contributes to functional independence and a more favorable perception of health status [34].

In addition, the observed differences may be related to sample characteristics, relatively small sample size, clinical heterogeneity of the study population, disease severity, presence of comorbidities, and variability in nutritional status [35]. It is also possible that in patients with more pronounced cachexia, sarcopenia, or severe COPD, associations between phase angle, adiposity-related parameters, and quality of life might become more evident [36].

Taken together, the present findings highlight the importance of fat-free and skeletal muscle mass parameters in the assessment of quality of life in patients with COPD. These parameters may represent more functionally relevant indicators than isolated adiposity-related characteristics [37]. At the same time, the findings regarding phase angle should be interpreted cautiously and require further investigation, as its clinical significance may be more closely related to overall somatic, nutritional, and prognostic status rather than to a direct association with quality of life [38].

### Strengths and Limitations

This study was exploratory in nature and does not allow conclusions regarding causal relationships or temporal associations. The sample size was moderate, and the cohort consisted predominantly of male participants, which may limit the generalizability of the findings to broader COPD populations.

The present analysis primarily focused on bioimpedance-derived body composition parameters and quality-of-life outcomes. Future studies involving larger cohorts may expand upon these findings by incorporating additional clinical variables, including smoking status, comorbidities, exacerbation history, pharmacological treatment, nutritional status, handgrip strength or muscle strength, and disease severity.

Prospective longitudinal studies may also help clarify the temporal relationship between changes in body composition and quality-of-life outcomes in patients with COPD.

## 5. Conclusions

In the present study, patients with COPD demonstrated increased body fat mass despite a median BMI within the normal weight range, whereas fat-free mass and skeletal muscle mass showed the most consistent associations with the physical and psychological domains of the WHOQOL-BREF.

These findings suggest that bioelectrical impedance analysis may serve as an additional tool that expands the assessment of patients with COPD beyond the traditional use of BMI alone. Evaluation of the relationship between fat mass, fat-free mass, and skeletal muscle mass may help identify unfavorable body composition alterations potentially associated with functional status and subjective health perception.

Thus, the results of the present study highlight the importance of a more comprehensive approach to body composition assessment in patients with COPD. Further prospective studies with larger sample sizes are required to clarify the clinical significance of these parameters and their potential role in monitoring the condition of patients with COPD.

## Figures and Tables

**Table 1:** Demographic and clinical characteristics, bioimpedance-derived body composition parameters, and quality-of-life indicators in patients with COPD (n = 75).

Variable	Value	Reference Values
Demographic and clinical characteristics		
Age (years)	54.9 ± 7.1	
Height (cm)	171.0 ± 8.5	
Weight (kg)	66.9 (60.3–85.0)	
BMI (kg/m^2^)	23.4 (20.8–28.9)	18.5–24.9
Body composition parameters		
Body fat mass, BFM (kg)	17.6 (10.8–26.9)	
Fat-free mass, FFM (kg)	51.4 (44.8–57.3)	
Skeletal muscle mass, SMM (kg)	28.3 (24.0–31.5)	
Percent body fat, PBF (%)	26.6 (17.4–33.6)	
Percent body fat, PBF (%) male	24.7 (15.8–31.8)	male 10–20%
Percent body fat, PBF (%) female	38.7 (33.9–43.6)	female 18–28%
ECW/TBW ratio	0.386 (0.382–0.391)	0.36–0.39
Basal metabolic rate, BMR (kcal/day)	1480.5 (1337–1608)	
Visceral fat area, VFA (cm^2^)	86.7 (54.1–133.9)	<100
Bone mineral content, BMC (kg)	2.83 (2.47–3.25)	
Phase Angle (50 kHz)		
Whole-body PhA (°)	5.1 (4.5–5.6)	
Whole-body PhA (°) male	5.2 (4.7–5.8)	male ~5.0–7.0
Whole-body PhA (°) female	4.6 (4.2–5.1)	female ~4.5–6.5
Right arm PhA (°)	5.2 (4.6–5.7)	
Left arm PhA (°)	5.1 (4.4–5.5)	
Trunk PhA (°)	7.3 (6.6–8.0)	
Right leg PhA (°)	4.8 (4.1–5.2)	
Left leg PhA (°)	4.8 (4.2–5.2)	
Pulmonary function		
FVC (%)	72.0 (55.1–92.9)	
FEV1 (%)	45.1 (32.2–63.4)	
FEV1/FVC (%)	55.0 (38.7–68.6)	
Quality of life (WHOQOL-BREF)		
Physical health (QoL1)	20 (18–22)	
Psychological (QoL2)	19 (17–21)	
Social relationships (QoL3)	10 (9–12)	
Environment (QoL4)	25 (23–28)	

Reference values are provided for parameters with established clinical threshold ranges. Body composition parameters, including body fat mass (BFM), fat-free mass (FFM), skeletal muscle mass (SMM), bone mineral content (BMC), and segmental phase angle, are presented descriptively, as their absolute values depend on individual anthropometric characteristics of the participants.

**Table 2:** Multivariable linear regression analysis including body composition parameters (n = 75).

*QoL1 (Physical)*			
**Predictor**	**B**	**β**	***p*** **-value**
*Intercept*	*19.10*	*—*	*<0.001*
FFM	0.10	0.35	**0.031**
BMR	0.003	0.22	0.102
VFA	0.017	0.14	0.271
BMC	0.89	0.24	0.108
**Predictor**	**B**	**β**	***p*** **-value**
*Intercept*	*19.84*	*—*	*<0.001*
SMM	0.12	0.33	**0.036**
BMR	0.004	0.25	0.082
VFA	0.019	0.16	0.238
BMC	0.93	0.25	0.096
*QoL2 (Psychological)*			
**Predictor**	**B**	**β**	***p*** **-value**
*Intercept*	*16.82*	*—*	*<0.001*
FFM	0.11	0.34	**0.029**
BMR	0.004	0.24	0.087
VFA	0.012	0.12	0.312
BMC	1.02	0.29	**0.041**
**Predictor**	**B**	**β**	***p*** **-value**
*Intercept*	*17.46*	*—*	*<0.001*
SMM	0.14	0.36	**0.026**
BMR	0.005	0.27	0.072
VFA	0.014	0.14	0.268
BMC	1.05	0.30	**0.038**
*QoL3 (Social)*			
**Predictor**	**B**	**β**	***p*** **-value**
*Intercept*	*9.84*	*—*	*<0.001*
FFM	0.04	0.18	0.214
BMR	0.002	0.15	0.276
VFA	−0.009	−0.11	0.338
BMC	0.52	0.19	0.187
**Predictor**	**B**	**β**	***p*** **-value**
*Intercept*	*10.12*	*—*	*<0.001*
SMM	0.05	0.17	0.228
BMR	0.002	0.16	0.249
VFA	−0.010	−0.12	0.312
BMC	0.55	0.20	0.173
*QoL4 (Environment)*			
**Predictor**	**B**	**β**	***p*** **-value**
*Intercept*	*453.59*	*—*	*0.104*
FFM	25.05	61.66	0.123
BMR	−1.16	−61.69	0.123
VFA	0.011	0.146	0.284
BMC	0.36	0.048	0.940
**Predictor**	**B**	**β**	***p*** **-value**
*Intercept*	*2.83*	*—*	*0.906*
SMM	−1.58	−2.339	0.379
BMR	0.047	2.488	0.375
VFA	0.011	0.147	0.286
BMC	−1.06	−0.140	0.829

Italic text indicates WHOQOL-BREF domains, while bold text indicates column headings. B—unstandardized regression coefficient; Statistically significant predictors are shown in bold (*p* < 0.05). FFM—fat-free mass; BMR—basal metabolic rate; VFA—visceral fat area; BMC—bone mineral content; SMM—skeletal muscle mass.

## Data Availability

The data presented in this study are available from the corresponding author upon reasonable request. The data are not publicly available due to privacy and ethical restrictions.

## Supplementary Materials

The following supporting information can be downloaded at: https://www.mdpi.com/article/10.3390/jcm15103839/s1, Table S1: Spearman correlation analysis between quality-of-life domains and bioimpedance-derived body composition parameters.

## Abbreviations

The following abbreviations are used in this manuscript:

COPD	Chronic Obstructive Pulmonary Disease
QoL	Quality of Life
WHOQOL-BREF	World Health Organization Quality of Life-BREF
BMI	Body Mass Index
BIA	Bioelectrical Impedance Analysis
DSM-BIA	Direct Segmental Multi-frequency Bioelectrical Impedance Analysis
PhA	Phase Angle
FFM	Fat-Free Mass
SMM	Skeletal Muscle Mass
BFM	Body Fat Mass
PBF	Percent Body Fat
VFA	Visceral Fat Area
ECW/TBW	Extracellular Water-to-Total Body Water Ratio
BMC	Bone Mineral Content
BMR	Basal Metabolic Rate
FEV_1_	Forced Expiratory Volume in One Second
FVC	Forced Vital Capacity
GOLD	Global Initiative for Chronic Obstructive Lung Disease
CRQ	Chronic Respiratory Questionnaire
CAT	COPD Assessment Test
SGRQ	St. George’s Respiratory Questionnaire
LCOPD	Living with COPD Questionnaire
VIF	Variance Inflation Factor
SD	Standard Deviation
