# Supplementary material for: Association Between Phase Angle, Muscle Mass Distribution, and Quality of Life in Patients with Chronic Obstructive Pulmonary Disease
Source: J Clin Med. 2026 May 16;15(10):3839. doi: 10.3390/jcm15103839 (PMC13207835; doi:10.3390/jcm15103839)
Supplement: Supplementary file 1 [file jcm-15-03839-s001.zip › jcm-4258592-supplementary.pdf]

Table S1. Spearman correlations between WHOQOL-BREF domains and BIA parameters (n = 75)

Variable	Domain	Spearman r	p-value
**BMI**	Physical	0.199	0.089
	Psychological	0.190	0.104
	Social	0.030	0.797
	Environmental	0.109	0.356
**BFM**	Physical	0.201	0.085
	Psychological	0.106	0.368
	Social	-0.036	0.761
	Environmental	0.095	0.421
**FFM**	Physical	0.138	0.241
	Psychological	0.238	**0.041**
	Social	0.131	0.265
	Environmental	0.080	0.495
**SMM**	Physical	0.130	0.271
	Psychological	0.240	**0.040**
	Social	0.123	0.298
	Environmental	0.070	0.555
**PBF**	Physical	0.173	0.140
	Psychological	0.062	0.600
	Social	-0.085	0.473
	Environmental	0.080	0.499
**ECW/TBW**	Physical	0.094	0.427
	Psychological	-0.196	0.094
	Social	0.099	0.399
	Environmental	0.092	0.434
**BMR**	Physical	0.138	0.242
	Psychological	0.236	**0.043**
	Social	0.129	0.272
	Environmental	0.078	0.507
**VFA**	Physical	0.223	0.056
	Psychological	0.098	0.405
	Social	-0.061	0.607
	Environmental	0.109	0.353
**BMC**	Physical	0.202	0.085
	Psychological	0.249	**0.033**
	Social	0.097	0.411
	Environmental	0.094	0.426
**Whole-body PhA**	Physical	-0.120	0.308
	Psychological	0.136	0.249
	Social	-0.075	0.526
	Environmental	-0.093	0.429
**Right arm PhA**	Physical	-0.098	0.405
	Psychological	0.148	0.210
	Social	-0.061	0.607
	Environmental	-0.080	0.496
**Left arm PhA**	Physical	-0.105	0.374
	Psychological	0.152	0.196
	Social	-0.058	0.622

	Environmental	-0.072	0.542
**Trunk PhA**	Physical	-0.134	0.255
	Psychological	0.121	0.304
	Social	-0.082	0.488
	Environmental	-0.101	0.392
**Right leg PhA**	Physical	-0.112	0.340
	Psychological	0.139	0.238
	Social	-0.070	0.556
	Environmental	-0.089	0.450
**Left leg PhA**	Physical	-0.118	0.317
	Psychological	0.141	0.231
	Social	-0.066	0.574
	Environmental	-0.084	0.476
